# Towards An Advanced Graphene-Based Magnetic Resonance Imaging Contrast Agent: Sub-acute Toxicity and Efficacy Studies in Small Animals

**DOI:** 10.1038/srep17182

**Published:** 2015-12-02

**Authors:** Shruti Kanakia, Jimmy Toussaint, Dung Minh Hoang, Sayan Mullick Chowdhury, Stephen Lee, Kenneth R. Shroyer, William Moore, Youssef Z. Wadghiri, Balaji Sitharaman

**Affiliations:** 1 Department of Biomedical Engineering, Stony Brook University, New York, NY USA; 2 Center for Advanced Imaging Innovation and Research (CAI^2^R), New York University School of Medicine, New York, NY USA; 3 Bernard and Irene Schwartz Center for Biomedical Imaging, Department of Radiology, New York University School of Medicine, New York, NY USA; 4 Department of Pathology, Stony Brook University, Stony Brook, NY, USA; 5 Department of Radiology, Stony Brook University, Stony Brook, NY, USA

## Abstract

Current clinical Gd^3+^-based *T*_*1*_ magnetic resonance imaging (MRI) contrast agents (CAs) are suboptimal or unsuitable, especially at higher magnetic fields (>1.5 Tesla) for advanced MRI applications such as blood pool, cellular and molecular imaging. Herein, towards the goal of developing a safe and more efficacious high field *T*_*1*_ MRI CA for these applications, we report the sub-acute toxicity and contrast enhancing capabilities of a novel nanoparticle MRI CA comprising of manganese (Mn^2+^) intercalated graphene nanoparticles functionalized with dextran (hereafter, Mangradex) in rodents. Sub-acute toxicology performed on rats intravenously injected with Mangradex at 1, 50 or 100 mg/kg dosages 3 times per week for three weeks indicated that dosages ≤50 mg/kg could serve as potential diagnostic doses. Whole body 7 Tesla MRI performed on mice injected with Mangradex at a potential diagnostic dose (25 mg/kg or 455 nanomoles Mn^2+^/kg; ~2 orders of magnitude lower than the paramagnetic ion concentration in a typical clinical dose) showed persistent (up to at least 2 hours) contrast enhancement in the vascular branches (Mn^2+^ concentration in blood at steady state = 300 ppb, per voxel = 45 femtomoles). The results lay the foundations for further development of Mangradex as a vascular and cellular/ molecular MRI probe.

Magnetic resonance imaging (MRI) is one of the most powerful clinical imaging techniques in diagnostic medicine that non-invasively renders exquisite anatomical details of soft and hard tissues for improved diagnosis of many pathologies. In clinical MRI, the nuclear magnetic resonance (NMR) signals from water protons in living tissues are used to image organs and disease sites in three dimension (3D) ranging from brain lesions and tumors throughout the body to myocardial infarctions^1^,^2^. Conventional MR signal intensity can be modulated by manipulating three very important intrinsic tissue factors: proton density, longitudinal relaxation time *T*_*1*_, and transverse relaxation time *T*_*2*_. Various imaging pulse sequences have been developed to highlight the endogenous differences in *T*_*1*_ or *T*_*2*_ relaxation times of bodily fluids and tissues to enhance contrast^1^. In addition, MRI contrast agents (CAs) are routinely employed as exogenous compounds during MRI examinations to enhance tissue contrast, and according to various estimates, globally ~30–44% of the >50 million clinical procedures use MRI CAs^2^.

Gadolinium (Gd^3+^)-based small molecule complexes dominate the MRI CA market (>95% market share, >$1 Billion market in 2012)^3^, and are considered the “gold standard.” The majority of current clinical Gd^3+^-based MRI CAs are considered as “first pass” agents as they are detectable by MRI within the first few minutes in the circulatory system after injection to rapidly extravasate and are subsequently eliminated via the kidneys^4^. However, these MRI CAs, due to their short-lived blood recirculation, offer a narrow imaging window that are suboptimal for Magnetic Resonance Angiography (MRA)^5^, widely employed to detect vascular abnormalities associated with aneurysm, hemorrhage, embolism, and stroke. Due to heart and respiratory movements, contrast enhanced MRA requires a significant decrease in blood *T*_*1*_^6^. This decrease is typically achieved by rapid injection of a large dose of clinical first pass agent MRI CAs such as Magnevist^6^. However, the higher dose injection protocol results in a very sharp bolus with marked *T*_*1*_ variations in image acquisition of the k-space sampling, causing image artifacts^7^,^8^. Moreover, previous MRI studies indicate that contrast between the arteries and surrounding tissue can significantly decrease due to extravasation of the CA^5^. To overcome these challenges, Gd^3+^-macromolecule complexes have been designed as advanced intravascular or blood pool agents with longer blood half-life and slower extravasation into the interstitial space compared to first-pass agents to first-pass agents. One agent (Ablavar® (Gadofosveset Trisodium)^9^,^10^, previously marketed under the trade names of MS-325 and Vasovist) is clinically approved to evaluate aortoiliac occlusive disease in adults with known or suspected peripheral vascular disease^11^. However, studies indicate that *r*_*1*_ relaxivities (an important measure of imaging efficacy defined as change in the relaxation rate (R_1 _= 1/*T*_*1*_ s^−1^) of the water protons per unit concentration of CA (mM)) of current clinical MRI CAs, with minimum detectable concentration of tens of μM to provide satisfactory contrast enhancement, are suboptimal for advanced MRI-based molecular and cellular imaging applications^12^,^13^. Theoretical studies also postulate the possibility to design novel MRI CAs with relaxivities (depending on the magnetic field) one to two orders of magnitude greater than current clinical agents^14^,^15^. Additionally, more and more MRI installations operate at higher magnetic fields (>1.5 Tesla) where Gd^3+^-based small molecule “first pass” agent or large molecule blood pool MRI CAs show significant drop in relaxivity^15^. Furthermore, some (Gd^3+^)-based clinical CAs have been reported to induce nephrogenic systemic fibrosis (NSF) in patients with moderate to severe renal insufficiency and vascular or metabolic disorders^16^,^17^,^18^. Thus, a safe and more efficacious *T*_*1*_ MRI CA that allows significant contrast enhancement at substantially lower dosages than (Gd^3+^)-based clinical CAs for advanced applications, such as blood pool or cellular or molecular imaging, would constitute a significant advancement.

Other than gadolinium, the elements manganese and iron have also been employed in the design of *T*_*1*_ and *T*_*2*_ MRI CAs, respectively^19^,^20^. Manganese especially has garnered attention as a possible alternative to gadolinium^20^. In fact, it was the first paramagnetic element suggested for enhancing imaging contrast in a phantom by Lauterbur in his landmark paper in 1973^21^. It was subsequently tested successfully on differentiating tissue contrast from various organs by the same group^22^. Yet, only one chelated form, manganese dipyridoxyl diphosphate (Mn-DPDP (brand name:Teslascan); Mangafodipir Trisodium) is FDA approved for clinical use^23^.

Nanoparticle-based *T*_*1*_ MRI CAs, including complexes of gadolinium with carbon nanostructures such as fullerenes (gadofullerenes), carbon nanotubes and graphene, have been proposed as *T*_1_ CAs^15^,^20^,^24^,^25^,^26^,^27^,^28^,^29^,^30^. Most of these studies have reported the *in vitro* relaxivity of these complexes at various magnetic fields. A few studies have also reported proof-of-principle *in vivo* efficacy at high magnetic fields (>1.5 Tesla) after systemic injection into normal rodents or direct tumor injection in rodent models of cancer. In the case of gadolinium carbon nanostructure complexes, these studies have only been reported with gadofullerenes^25^. However, no information is available regarding the acute/subacute toxicity, maximum tolerable doses (MTD) of these complexes, or assessment of other important issues such as respiratory and cardiovascular pharmacology safety. Consequently, the therapeutic indices or potential diagnostic dosages of these formulations remain unknown.

Recently, we reported the synthesis and characterization of graphene nanoparticles (a.k.a. graphene nanoplatelets) intercalated with manganese (Mn^2+^) ions as novel *T*_*1*_ MRI CAs^31^. The nanoparticles upon non-covalent functionalization with FDA-approved natural polymer dextran (weight ratio of GNP: dextran = 3:2), are disc-shaped (average diameter and thickness of 100 nm and 3 nm, respectively)^32^,^33^,^34^, and hereafter referred as Mangradex. Representative transmission electron microscope (TEM) image of Mangradex nanoparticles and digital image of the formulation are included in the supplementary information (Figure S1). We have previously assessed the *in vitro* physicochemical properties of Mangradex^32^,^34^, and *in vivo* small animal intravenous (IV) dose range-finding, expanded acute toxicity and pharmacokinetics^33^, as well as performed *in vitro* and *in vivo* small animal studies to characterize its hematological and vasoactive effects^34^. Table S1 and S2 summarizes experiments and salient results of those studies. Our *in vitro* relaxometry and phantom MRI results demonstrate that Mangradex exhibits relaxivity values ~20 fold greater (92 mM^−1^S^−1^) than current clinical contrast agents (~4.5 mM^−1^S^−1^)^32^ and could significantly enhance MR contrast at lower dosages. Previously reported dose ranging and expanded acute toxicity studies, at doses between 1 mg/kg to 500 mg/kg, showed a dose-dependent toxicology and pharmacokinetics, indicated that the maximum tolerated dose (MTD) for Mangradex was 50 mg/kg, < MTD < 125 mg/kg^33^, and a majority of the nanoparticles were excreted within 24 h. In this study, we report the outcomes of safety studies of Mangradex upon sub-acute IV administration in normal healthy rats, and efficacy studies after single IV administration of Mangradex in wild type mice at 7 Tesla (7T) magnetic field strength.

## Results

### Subacute toxicity

Table 1 summarizes the various groups and any mortality observed for the sub-acute toxicity study. During the 3-week multiple-dose (3 times per week) toxicity study in rats, mortality was observed in 2 out of 8 animals (1 M and 1 F) at 100 mg/kg dose during the 2^nd^ week; a male rat deceased after 5 injections and a female rat after 4 injections. Necropsy performed on these animals by in-house veterinary technician showed that the lungs were darker than usual suggesting presence of nanoparticles. All other major organs heart, liver, kidneys and brain were normal without any treatment related adverse effects. From the surviving animals, 1 male rat injected with 100 mg/kg dose elicited response at the injection site in the form of mild local swelling during the 3^rd^ week after 6 injections, this persisted throughout the end of the study. In all other surviving animals, no other treatment related adverse effects were observed. Additionally, in these animals, there were no changes in the animal posture or behavior with cage mates at any of the injected doses of Mangradex. Further, both male and female rats at all dose groups did not show any treatment related changes in terms of body weights, breathing, blood pressure and heart rate as shown in Figures S2–S4.

### Histology

**Figure 1:**
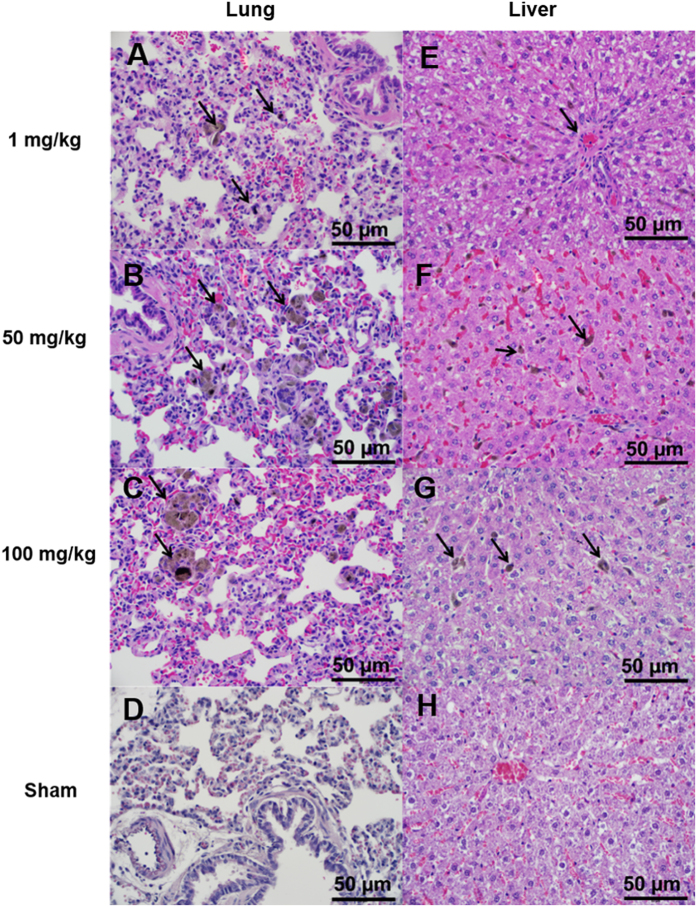
Representative high (400X) magnification photomicrographs illustrating pulmonary and hepatic histology from chronic study animals at 1 mg/kg, 50 mg/kg, 100 mg/kg Mangradex and sham. **Lung** (**A**) at 1 mg/kg, (**B**) at 50 mg/kg and (**C**) at 100 mg/kg- with pigment (arrows) within alveolar macrophages suggestive of the presence of graphene nanoparticles, (**D**) sham - without diagnostic abnormality. **Liver** – (**E**) at 1 mg/kg - showing minimal steatosis, but without diagnostic abnormality, (**F**) at 50 mg/kg- showing pigmented macrophage in Kupffer cells suggestive of graphene particles. No sign of inflammation, (**G**) at 100 mg/kg - more pigmented than at lower dose, (**H**) sham - without diagnostic abnormality.

Tissue histology of major organs (brain, heart, liver, lung, kidney, spleen) was performed on sham and experimental animals that survived the entire 3 week duration of the study. Figure 1 shows representative histologic tissue sections at 400X magnification settings from sham and rats injected with 1, 50 or 100 mg/kg Mangradex 9 times over 3 weeks. The most common microscopic findings were the presence of pigment in hepatic Kuppfer cells and in pulmonary alveolar macrophages, consistent with the presence of graphene nanoparticles. Overall, more pigment was observed at higher dosages (50 and 100 mg/kg) in the lungs than at the lower dose. There were no treatment related effects in brain, heart, spleen and kidney. In all dose groups, no histological evidence of inflammation was observed in any tissue.

### Blood analysis

A complete blood count (CBC) and established serum biochemistry assays (comprehensive lipid and metabolic panel) were performed and analyzed for the 1, 50 and 100 mg/kg doses of Mangradex, dextran, mannitol and sham. The results of the experimental groups were compared with sham, mannitol and dextran group, and also with the normal range of blood parameters of Wistar rats (information provided by the vendor, Charles River Laboratories, Wilmington, MA, USA)^35^. Table 2 displays various hematological parameters with values outside the normal range. All other parameters with values either within the normal range are displayed in Table S3. Indicators of kidney function- blood urea nitrogen (BUN) and creatinine (CRE) were normal (Table S3). Out of the five important hepatic indicators - alkaline phosphatase (ALP), total protein (TP), albumin (ALB), alanine transaminase (ALT), and aspartate transaminase (AST); ALT and ALP levels were elevated in the experimental groups as well as in sham and control groups. Blood glucose (GLU) levels were above the normal range in sham, mannitol, 1 mg/kg (F) and 50 mg/kg (M) treatment group and Triglycerides (TRIG) levels were above the normal range in sham, dextran and 1 mg/kg (M) treatment group (Table 2). All other parameters were in healthy normal range (Table S3).

### Imaging efficacy

**Figure 2:**
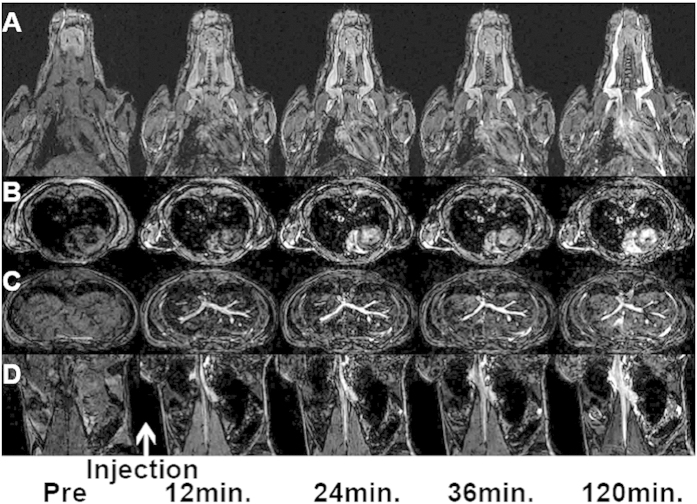
Three-dimensional (3D) imaging of the whole mouse body in less than 12-minutes using a 7T mouse MRI scanner equipped with a homemade RF coil enabling serial imaging with 200-μm isotropic spatial resolution. The example of image dataset shown compares different organ and body regions prior and following single injection of Mangradex at 25 mg/kg. (**A**) the upper body including the head, neck, heart and lungs; (**B**) slice re-orientation obtained from the same lung and heart area described in (**A**); (**C**) the section covering the lung and liver region; (**D**) a coronal view from the lower abdominal region that includes the kidneys and the spleen.

Figure 2 shows the whole body coverage achieved using a 7T mouse MRI after single injection of Mangradex (Dose = 25 mg/kg Mangradex or 455 nanomoles Mn^2+^ ions/kg). With the level of anatomical details achievable by our MR imaging protocol (200-μm isotropic resolution) in combination with our homemade MRI probe enabling full mouse body coverage, we were able to examine most of the main organs and the major vascular branches. The results from the serial MRI performed in contrast agent-free subjects followed by serial acquisitions at 12, 24, 36, 48, 60, 72, 84, 96, 108 and 120 minutes post-injection in all mice clearly demonstrated the blood-pool nature of our compound Mangradex. As illustrated by the example shown in Fig. 2A, the intravascular space is difficult to discern prior to the injection of the contrast agent (depicted by the column “Pre”) throughout the different parts of the mouse body. Each row ranging from **A** to **D** corresponds to either coronal (A&D) or axial (B&C) sections at various levels of the mouse body as follow: A) covers the upper body including the head, neck, heart and lungs; B) is an axial slice re-orientation obtained from the same lung and heart area described in A) while in C) the section covers the lung and liver region; D) is a coronal view from the lower abdominal region that includes the kidneys and the spleen. As evidenced by the example shown, there is an obvious signal enhancement starting from the first image set acquired 12-min. post-injection. The greatest tissue contrast emanated from the vascular space in the various regions of the body as well as from the cardiac chambers seen in row A) with a long axis view of the heart & B) through its short axis section. This tissue contrast was most notable within the large vascular branching: head & neck in row A), liver in row C) as well as highlighting the abdominal aorta and inferior vena cava in row D). This enhancement was maintained throughout the two-hour period in which all the subjects were examined every 12-min. and where only a subset of the imaging time points corresponding to 12-, 36- and 120-min. post-injection are shown in Fig. 2A. Surprisingly, the kidneys experienced a dark enhancement that was immediate following the injection and was maintained throughout the imaging session as seen on row **D**).

**Figure 3:**
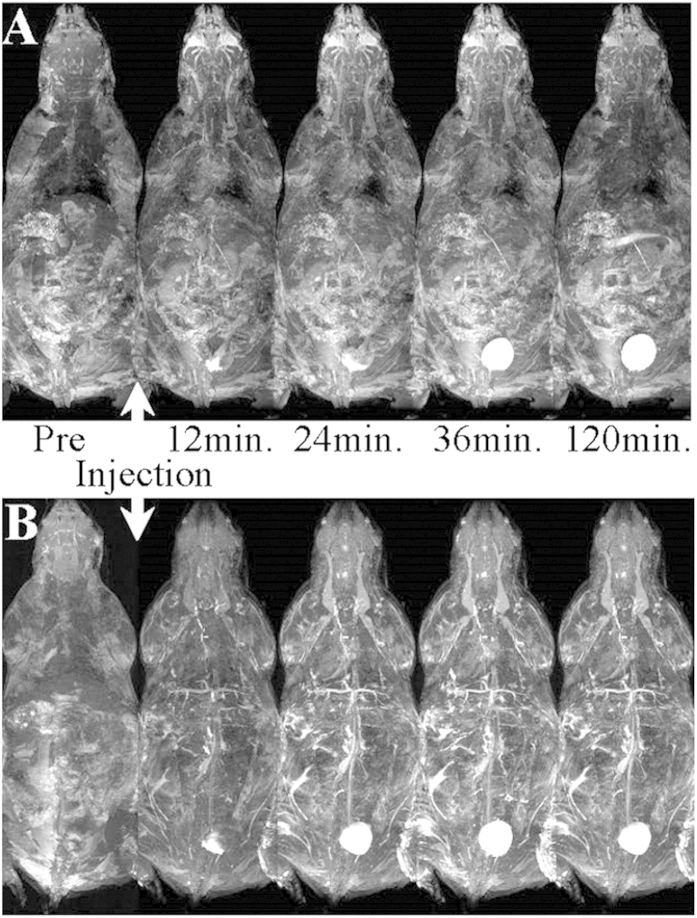
3D visual rendering of the whole mouse body throughout the time course of this study via maximum-intensity-projections to facilitate the qualitative comparison of the bio-distribution and pharmacokinetics between CA injected in two groups. The effects of the contrast are illustrated as follow: (**A**) Pre- & post- injection of Ablavar® (455 nmoles/kg of Gd^3+^) as well as (**B**) pre- & post Mangradex injection at 25 mg/kg (equivalent to 455 nmoles/kg of Mn^2+^).

The qualitative comparison of image contrast efficacy and pharmacokinetics was assessed using Maximum-Intensity-Projection of the serial 3D image datasets and by identifying regions of MRI signal enhancement induced by the presence of either Mangradex or the clinical blood pool agent Ablavar® at the prescribed clinical dose (Gd^3+^ ion concentration = 455 nanomoles/kg). Figure 3 illustrates an example from each mouse group corresponding to the same imaging time window displayed in Fig. 2 (12-, 36- and 120-min post-injection). While subtle tissue contrast changes can be seen gradually enhancing in the major vessel branches immediately following the injection of Ablavar (Fig. 3 row **A**), these signal enhancements gradually decayed (data not shown) and vanished by the last imaging time point corresponding to 120-min. Expectedly, the kidneys demonstrated immediate and subtle bright enhancement consistent with the renal excretion known to Ablavar following injection. The subtle increase in signal from kidneys can be partially distinguished in Fig. 3, row A which could be likely overshadowed when using the MIP algorithm, by superficial tissue and vessel enhancement. On the other hand, the injection of Mangradex at equivalent concentrations demonstrated an immediate and more pronounced contrast enhancement of the vasculature that persisted throughout the 2-hour imaging examination (Fig. 3**, row B**). Importantly, the injection of either Mangradex or Ablavar resulted in gradual and consistent enhancement of the bladder as shown in Figure 3, **row A&B**.

**Figure 4:**
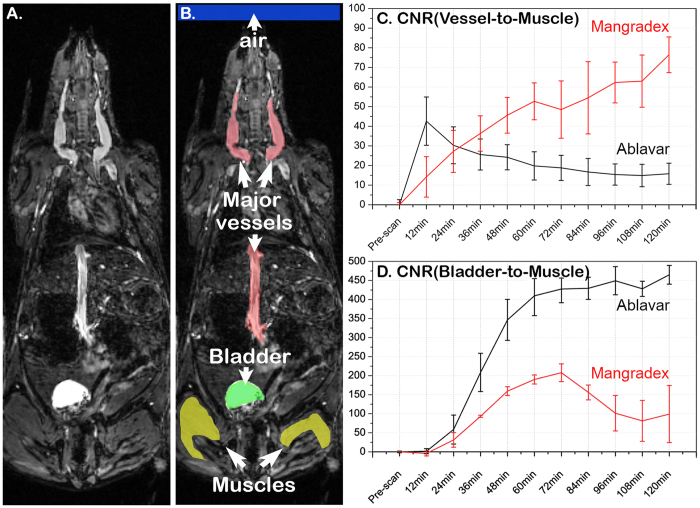
*In vivo* quantitative comparison of the image contrast efficacy and enhancement kinetics of Mangradex with the clinical blood pool agent Ablavar® in C57Bl6 wild type mice. (**A**) Example of a horizontal section covering the whole mouse body in which the presence of contrast agent eases the identification of the major vessels as well as the bladder where the paramagnetic ligand is excreted. (**B**) ROIs of the most important parts of the mouse body relevant to this study were examined in order to measure the SNRs and the corresponding CNRs including the large vessel (red area), the bladder (green area), the hind limb muscles (yellow area) as well as the background noise (large blue area at the top). (**C**) Shows the contrast preeminence and the sustained effect of Mangradex (red plot) assessed in large vessels compared to the clinical agent Ablavar during serial imaging analysis. (**D**) A Similar time-curve was also established in order to examine and compare the apparent excretion of both agents expected through the bladder suggesting that Mangradex is longer lived and progressively excreted.

The quantitative comparison was assessed from the serial 3D image datasets of the animal groups administered with either Mangradex or Ablavar® through a comparison of contrast-to-noise ratios (CNR) from regions easy to delineate anatomically thanks to the well-defined tissue contrast obtained and illustrated by the example in Figure 4A,B. The CNRs were calculated as the difference in signal-to-noise ratios (SNRs) of the major large vessels (depicted by the red area), the bladder (highlighted by the green area) and the background noise (blue square) relative to the hind limb muscles (depicted by the yellow area). Figure 4C represents the plots summarizing the vessel-to-muscle CNRs measurements from both Mangradex (red line) and Ablavar (black line) groups while Figure 4D corresponds to the results obtained from the bladder-to-muscle CNRs. Both quantitative measurements demonstrate the greater and sustained contrast enhancement of Mangradex compared to Ablavar. Quantitative comparison on tissue contrast was also assessed between both agents by calculating signal-to-noise ratios (SNRs) and contrast-to-noise ratios (CNRs) in various regions of interest (ROI) including the major large vessels throughout the whole body, the bladder and muscle.

## Discussion

The overall objectives of this study were (1) to identify the no-observed adverse effect level (NOAEL) within the previously identified maximum tolerated dose (MTD) range that could be used to help identify the potential diagnostic dose, and (2) to evaluate its efficacy as an MRI CA in healthy mice at a potential diagnostic dose. Our previous *in vitro* physio-chemical characterization studies show that this nanoparticle formulation is hydrophilic, thermally stable under physiological conditions, and when mixed with the excipient mannitol, iso-osmolar and iso-viscous to blood, and forms stable colloidal dispersions in deionized water and biological fluids (buffers and blood) up to 100 mg/ml concentrations^32^.

FDA and International Conference on Harmonization (ICH) guidelines on preclinical safety studies of MRI CAs have also emphasized that repeat dose chronic toxicity studies be conducted to identify potential therapeutic dosages for first-in-human trials^36^,^37^. These studies are considered important since they reliably provide information not easily predictable by single dose acute toxicology studies; information such as histopathological changes upon repeated or chronic exposure of MRI CA and other cumulative effects. According to FDA guidelines, the recommended duration of chronic toxicity for new pharmaceutical development depends on its duration, therapeutic indication (MRI CA in this case), scope of the clinical trial and could range from 2 weeks to 9 months^37^,^38^,^39^. For FDA approved MRI CAs Gadodiamide (Omniscan)^40^, Mn-DPDP (Teslascan)^41^, and Gadoversatamide (Optimark)^42^ chronic toxicity studies were performed for 3 week duration with the CA administration frequency 3 times/week. Hence, for Mangradex the time points selected were 3 per week for three weeks at three escalating doses (low - 1 mg/kg, medium - 50 mg/kg, and high - 100 mg/kg). The maximum dose (100 mg/kg) selected was based on the outcomes of previous dose ranging, and expanded acute toxicity studies for Mangradex formulation^33^. In those studies, we found the maximum tolerated dose (MTD) for Mangradex was 50 ≤ MTD <125 mg/kg. Furthermore, neither any test article related adverse effects on the immune system nor any inflammatory response were noted after single IV injections at doses ≤100 mg/kg^33^. Hence, in this study, we have assessed chronic toxicity effects at doses ≤100 mg/kg. As the ratio of GNP: dextran in Mangradex is 3:2 by weight^32^, 40 mg/kg dose of dextran (equivalent of dextran by weight in 100 mg/kg of Mangradex) was selected as one of the controls. Mannitol (which was used to control osmolality of the Mangradex formulation) was selected as the second control.

The necropsy findings were consistent with acute toxicity results, where rats with mortality at higher dosages (>125 mg/kg) showed presence of Mangradex in pulmonary capillaries with symptoms similar to pulmonary congestion^33^. Additionally, the absence of distinguishable toxicity related changes in all major organs (heart, kidney, liver, spleen, and brain) suggest that there may be no secondary cause of death. Our previous biodistribution study showed that ~1–2% of the injected dose of Mangradex remained in lungs one day after single injection^33^. This result taken together with necropsy results suggests that after multiple injections, the nanoparticles that cannot be removed from the lungs may have accumulated in pulmonary capillaries impeding blood flow in microcirculation. In all surviving animals, at ≥50 mg/kg, the absence of Mangradex related histological changes in the brain, heart and spleen and minor histological changes (presence of brown granular pigment) noted in the liver and lung indicate that the cumulative effects of Mangradex does not adversely affect the major organs.

Mangradex related unfavorable reaction in the circulatory system can affect the immune system and/or hematological factors. Accordingly, standard serum chemistry test and complete blood panel analyses were performed. The results of blood chemistry parameters for Mangradex dosages and controls were compared with sham and published data by Charles River Laboratories^35^. Blood counts did not differ significantly in any dose groups. Elevated levels of hepatic function markers ALT and ALP are typically attributed to liver injury; however, since above normal values were observed in sham and controls as well, Mangradex treatment alone could not be attributed as the main reason for the measured elevated values. Similarly, increased levels of glucose and TRIG found in sham, mannitol or dextran were not dose dependent and elevation was noted in one of the experimental group. Thus, these increases cannot be extrapolated as solely Mangradex associated effects, and could be animal specific outcomes. Other blood chemistry parameters were within the normal healthy ranges and did not differ significantly between groups.

For the *in vivo* MRI studies, the choice of 25 mg/kg dose was based on the outcomes of all the *in vitro* and *in vivo* (including the repeat dose) safety studies^32^,^33^,^34^. Our previous studies indicate that, at concentrations equivalent to first pass of a bolus injection (1–50 mg/ml), the formulation neither elicits any significant allergic response of the immune system, nor induces pro-inflammatory endothelial dysfunction effects^33^,^34^. Similarly at potentially steady state equilibrium concentrations in blood (0.1–10 mg/ml), we found no protein binding or complement activation^32^,^34^. The maximum tolerated dose found from acute *in vivo* toxicity studies was 50 mg/kg ≤MTD < 125 mg/kg^33^ and based on the results of this study, the no-observed adverse effect level (NOAEL) was 50 mg/kg. Hence, potential therapeutic dose selected for *in vivo* MRI was less than half of the MTD. The clinical Gd^3+^-based blood pool agent Ablavar was used as control. The longer blood circulation of Ablavar compared to monomeric imaging agents such as Gd-DTPA or Gd-DOTA is attributed to its strong and high degree of reversible binding to serum albumin, while the blood circulation of Mangradex could be because of its hydrophilic properties, which is consistent with our previous *in vitro* studies showing that the partition coefficient of Mangradex is –0.18, suggesting that majority of the contrast agents remain in liquid phase than in lipid (tissue *in vivo*)^32^.

Numerous clinical studies have demonstrated the efficacy of Ablavar® in strongly enhancing blood vessels for MRA examinations^43^. We observed a similar vascular enhancement when injecting Ablavar® in mice and the sensitivity of our imaging setup enabled us to trace the recirculation of this blood pool agent for more than 1 hour (data not shown). For Mangradex, our findings from the *in vivo* rodent studies indicated immediate and very strong vascular signal contrast enhancement that was prolonged for more than 2-hours evidenced by the serial imaging depicted by the example shown in Figure 3, **row B**. Other covalently or non-covalently functionalized graphene formulations have shown analogous pharmacokinetics in mice following intravenous administration where sustained recirculation and excretion via the biliary and renal pathways were observed^44^,^45^. This route of clearance was consistent with our previous study^33^, and corroborated further by the darkening effect seen in the kidney (Fig. 2D). However, the unexpected signal blackening effect proved particularly puzzling when juxtaposed with the hyper-intense enhancement seen by the same particles in the recirculating blood prior to their renal excretion and subsequent downstream brightening when reaching the bladder. This immediate change in contrast effect when reaching the kidneys and later its positive contrast reversal may likely reflect an important change in the ratio R_2_ (R_2_ = 1/*T*_2_) to R_1_ (R_1_ = 1/*T*_1_) relaxivities of Mangradex. This R_2_/R_l_ alteration is commonly observed in iron oxide-based agents^46^, and varies with the intrinsic particle size but also reflects the aggregation or dispersion of magnetic particles when they react with surrounding tissue^47^. The degree of Mangradex aggregation and/or compartmentalization combined with the 7T magnetic field strength may be responsible for the signal “quenching” effect observed in the kidneys. The positive contrast enhancement obtained by Mangradex within the blood stream proved to be far superior to that provided with Ablavar® at equal molar concentration of paramagnetic agent evidenced by the comparative serial imaging shown in Figs 3 and 4. More importantly, the enhancement was achieved at a low Mn^2+^/kg dose of 455 nanomoles/kg; 66X and 220X lower than clinical dose of Ablavar® (Gd^3+^/kg dose = 0.03 mmol/kg^43^) and Magnevist (Gd^3+^/kg dose = 0.1 mmol/kg^7^), respectively. This dose is also in the range of average daily dietary intake of manganese (1.8–2.3 mg per day = 33–42 micromoles)^48^. At the Mn^2+^/kg dose used in this study, the estimated Mn^2+^ steady state blood concentration in mice (weight = 40 grams; total blood volume = 3.2 ml) after the first pass would be ~300 ppb or 45 femtomoles /voxel. Our previous relaxometric studies indicate that the dynamics of water within nanostructures with multiple layers of graphene and the interaction between water and Mn^2+^ ions in these confined spaces, affect the *T*_*1*_ relaxation mechanism, and could be responsible for the observed increase in contrast enhancement^31^. The molecular dynamics parameters that are modulated include the coordination number of inner-sphere water molecules (*q*) and water molecule residence lifetime (τ_M_) coordinated with the Mn^2+^ ions, and the rotational correlation time (τ_R_) of Mangradex. The combination of significant contrast enhancement at low detectable concentrations achieved at a high field strength taken together with the longer residence times in the blood introduces Mangradex not only as a great addition to the existing blood pool agent, but also suggests its suitability to be incorporated in the design of preclinical cellular/molecular imaging probes to monitor the progression of vascular pathologies.

## Materials and Methods

### Chronic toxicity

#### Animal care and dose ranges

All the experimental protocols were approved by and conducted in accordance with the guidelines of the Institutional Animal Care and Use Committee at Stony Brook University, NY. The 21 - day sub-acute toxicity study at three doses, was conducted in a total of 44 Wistar rats (male and female) weighing 200–250 g. Three groups of 8 rats (4 male and 4 female) had received Mangradex injections intravenously via the tail vein three times per week at the doses of 1 mg/kg, 50 mg/kg and 100 mg/kg of body weight. During injections the animals were anesthetized using isoflurane (1**–**2.5% mixture of O_2_/air, via inhalation). Control groups were given IV injection of mannitol (at the concentration of 55 mg/ml added to the Mangradex formulation to control the osmolality), and dextran (at the dose of 40 mg/kg, used for surface functionalization of GNP) with the same dosing regimen. Blood pressure and heart rate were measured using a non-invasive occlusion type tail cuff system (Coda 6, Kent Scientific System, PA) once per week after administration of Mangradex. The parameters evaluated included survival, clinical observations, body weight and diet observation. All the animals were sacrificed using 100% CO_2_ inhalation after 3 weeks and blood was obtained by cardiac puncture technique. All the major organs (heart, lungs, liver, brain, spleen, and kidneys) from each animal were harvested and prepared for histology analysis.

### Histology

Tissue samples were initially fixed in 10% formalin for 48 hours and were dissected into 3 mm segments. All organs were dissected symmetrically in the same manner to help ensure consistency. The tissues were then dehydrated in graded ethanol and paraffin-embedded. 5 μm sections were cut using a microtome and stained with Hematoxylin and Eosin (H&E) for histologic evaluation. Digital photomicroscopy was performed using a bright field microscope at 400X magnification. Histologic assessment of the tissue sections were performed by an experienced diagnostic and research anatomic pathologist (KRS).

### Blood analysis

From the total of 8 animals per dose of Mangradex, controls and sham, 6 animals (3 male, 3 female) were used for serum chemistry analysis. Animals were euthanized by 100% CO_2_ inhalation and ~8 ml blood was collected from each animal by cardiac puncture for blood analysis. Serum chemistry test and complete blood panel analyses were performed in a Clinical Laboratory Improvement Amendments (CLIA) certified facility at Stony Brook University Hospital.

### Statistical analysis

All data were analyzed for statistical significance by One-way analysis of variance (ANOVA) using Graphpad Prism (Graphpad software, Inc. La Jolla,, CA). The criterion for statistical significance was p ≤ 0.05.

### MRI Contrast agent preparation

For contrast enhanced-MRI studies, 10 mg/ml solution of Mangradex was prepared by dissolving 10 mg of lyophilized Mangradex powder in distilled deionized water. D-Mannitol (Sigma Aldrich, St Louis, MO) was added to make Mangradex solution iso-osmol to blood. The formulation was UV sterilized for 30 min. The dose for Mangradex per kg of mouse weight was 25 mg/kg or 455 nanomoles Mn^2+^ ion. The injection volume of Mangradex and clinically approved Ablavar (Lantheus Medical Imaging, Inc., N. Billerica, MA) was similar (50 μl). Ablavar diluted in saline dose per kg of mouse weight was 455 nanomoles Gd^3+^ ion.

### *In vivo* imaging

For imaging, C57BL/6 wild type female mice (Taconic farms Inc., Hudson, NY) with weight ranging from 25 g to 30 g were used. All mice were maintained in agreement with procedures approved by the Institutional Animal Care and Use Committee at New York University School of Medicine. Mice were anesthetized with isoflurane gas delivered via a vaporizer/anesthesia setup (VetEquip, Inc., Pleasanton, CA, USA): 5% isoflurane in air for induction followed by 0.5–1.5% isoflurane in air via nosecone for maintenance. Following anesthetic induction, each animal was catheterized via femoral artery to infuse the contrast agent using a polyethylene tubing PE-10 line (Intramedic, Becton Dickinson, Parsippany, NJ). Each subject was then placed supine and restrained on a custom made body holder bed with the cannula tubing extended long enough to enable remote infusion using a PHD-2000 computer-controlled syringe pump (Harvard Apparatus, Hollison, MA), while the animal was centered within the magnet bore. Body temperature was maintained between 35–37 °C with a warm air thermostat system while all basic physiologic signals (temperature and breathing rate) of the freely-breathing animals were monitored continuously (SA Instruments Inc., Stony Brook, NY). Respiratory motion was monitored using a pneumatic pillow fixed to the abdomen and the body temperature was measured via a temperature probe. The whole mouse bed was then subsequently inserted within a home-built radiofrequency (RF) coil. This circularly polarized MRI probe was developed in-house to resonate at a proton frequency of 300-MHz in both transmit and receive modes with dimensions (ID = 38-mm, L = 76-mm, AD = 36-mm) insuring a homogenous RF coverage of the adult whole mouse body.

MRI experiment were conducted using a 7-Tesla (7T) micro-MRI system consisting of a 7T 200-mm horizontal bore magnet (Magnex Scientific Ltd., Yarnton, UK) interfaced to a Bruker Biospec Avance-2 console (Bruker, Billerica, MA). The system was equipped with an actively shielded gradient coil (Resonance Research, Billerica, MA: BGA-9S; ID 90-mm, 750-mT/m gradient strength, 100-ms rise time). For *in vivo* micro-MRI (μ-MRI), a modified three-dimensional (3D) spoiled gradient echo (GE) sequence was used to acquire an additional self-gated signal on the readout dephasing gradient within each TR^49^. The gating signal was used retrospectively to generate artifact free image reconstruction sets with the following parameters: 200-μm isotropic spatial resolution; repetition time (TR) = 15 ms; bandwidth (BW) = 50 kHz, matrix = 384 × 128 × 128; field of view (FOV) = 76.8 mm × 25.6 mm × 25.6 mm; echo time (TE) = 6.2 ms, number of averages (N_AV_) = 3 and imaging time ~12 min. Flip angle (FA) = 34° was chosen to provide the greatest *T*_1_-enhancement contrast^50^. The advantage of the 3D-imaging approach with isotropic resolution is that the image set can be reprocessed in any desired slice orientation using NIH image J software (imagej.nih.gov/ij), facilitating image comparison during co-registration between animals acquired in separate sessions.

*T*_1_-weighted 3D-GE MRI scans of 12 min. duration each, were performed serially during every imaging session in mice to compare qualitatively the bio-distribution and pharmacokinetics of Mangradex (Dose = 25 mg/kg, equivalent to 455 nanomoles Mn^2+^ ions/kg body weight) with FDA-approved clinical intravascular agent Ablavar® (Gadofosveset trisodium). The protocol consisted of acquiring an initial image set serving as the contrast-free baseline followed by the infusion of the blood-pool contrast agent and serial scanning of nine image datasets at 12, 24, 36, 48, 60, 72, 84, 96, 108 and 120 minutes post-injection in all subjects.

### Image Analysis

In order to compare qualitatively the image contrast efficacy and enhancement kinetics of Mangradex with the clinical blood pool agent Ablavar®, 3D image datasets from both mouse groups were analyzed by generating Maximum-Intensity-Projection (MIP) throughout time course of the imaging session. In this approach, the MIP algorithm enables pixels with the highest value crossed via a chosen angle throughout the 3D image set to be projected for display on a 2D plane. This method permitted the visual rendering of the tissues where the contrast agent was distributed due to the signal enhancement it induced by its presence.

Quantitative comparison on tissue contrast was also assessed between both agents by calculating signal-to-noise ratios (SNRs) and contrast-to-noise ratios (CNRs) in various regions of interest (ROI) including the major large vessels throughout the whole body, the bladder and muscle. The SNR in each of these regions was calculated as SI/Stdev where, SI is the mean signal intensity within the ROI and Stdev is the standard deviation of the signal intensity within the background noise (air). The standard deviation of the noise was obtained by carefully choosing the largest possible ROI placed outside the subject in the image background while avoiding ghosting/aliasing or eye movement artifact regions.

The CNR was defined relative to the muscle region as (SI_(CA)_-SI_(muscle)_)/Stdev_(Noise)_ where, SI_(CA)_ is the mean signal intensity in the region where the contrast agent (CA) is localized in either the major vessels or in the bladder, SI_(muscle)_ is the mean signal intensity in the muscle region. Pre-contrast SNRs were subtracted from post-contrast SNRs to minimize differences in baseline signals from animal to animal. Similarly, pre-contrast CNRs were subtracted from post-contrast CNRs. SNRs and CNRs calculations were performed for each animal and then average values were reported for each group (n = 4).

## Conclusions

Mangradex formulations up to 50 mg/kg could serve as potential diagnostic doses. The formulation elicits dose-dependent presence of pigments in lungs and liver and no changes in brain, heart, spleen and kidney. The formulation at all dosages neither induces an inflammatory response in any tissue, nor causes any noticeable adverse effects in hematological (lipid panel, metabolic panel and serum chemistry) parameters. The formulations at low paramagnetic ion concentrations allow at high magnetic fields significant and sustained contrast enhancement of major vascular branches. Taken together, these results establish the basis for further employing Mangradex in studying vascular disease models and subsequently into higher species to facilitate its eventual clinical translation.

## Additional Information

**How to cite this article**: Kanakia, S. *et al*. Towards An Advanced Graphene-Based Magnetic Resonance Imaging Contrast Agent: Sub-acute Toxicity and Efficacy Studies in Small Animals. *Sci. Rep*. **5**, 17182; doi: 10.1038/srep17182 (2015).

## Supplementary Material

Supplementary Information

## Figures and Tables

**Table 1:** Mortality observed in animals at varying doses of Mangradex during the chronic toxicity study.

Group	Dose	Total Animals		No. of Dead animals	
		M	F	M	F
Mangradex	1 mg/kg	4	4	0	0
	50 mg/kg	4	4	0	1
	100 mg/kg	4	4	1*	1ψ
Control	Mannitol	3	3	0	0
Sham		3	3	0	0

Each animal received IV injections three times per week (Monday, Wednesday and Friday) for three weeks.

^*^Mortality on 7^th^ day after 4 injections of 100 mg/kg.

^ψ^Mortality on 10^th^ day after 5 injections of 100 mg/kg.

**Table 2:** Blood chemistry results for rats injected with Mangradex, dextran, or mannitol.

No.	Test	Sex	Normal	Sham	Mannitol	Dextran	1 mg/kg	50 mg/kg	100 mg/kg
A	ALT (U/L)	**M**	19–48	54.3 ± 7.5*	54.5 ± 2.1*	47.7 ± 7.0	73.3 ± 35.3*	43 ± 6.0	60.3 ± 13.5*
		**F**	14–64	54.5 ± 19.3	50.0 ± 7.2	53 ± 6.2	38.5 ± 0.7	45.8 ± 11.9	46.0 ± 11.0
B	ALP (U/L)	**M**	36–131	147.3 ± 45.5*	134 ± 32.5*	114.33 ± 8.4	161.3 ± 44.2*	138.8 ± 11.7*	119 ± 13.9
		**F**	18–62	75.3 ± 15.9*	89.3 ± 21.3*	71.3 ± 13.6*	93.5 ± 2.1*	88.8 ± 25.1*	97.0 ± 12.2*
C	Glucose (mg/dl)	**M**	116–184	220.6 ± 97.2*	207 ± 98.9*	154.11.3	181 ± 32.9	215.7 ± 30.7*	161.7 ± 37.7
		**F**	89–163	249.3 ± 65.9*	211 ± 81.4*	154 ± 16.7	214 ± 29.7*	183.3 ± 29.6	143.7 ± 7.4
D	TRIG (mg/dL)	**M**	27–160	215.7 ± 76.4*	137.5 ± 0.7	225.3 ± 65.7*	186.8 ± 55.2*	128.8 ± 48.9	141.8 ± 61.3
		**F**	16–175	93.3 ± 53.1	65 ± 18.9	69.7 ± 17.5	104 ± 16.9	68.8 ± 17.9	70 ± 40.6

Also included are sham controls. The data are shown as mean values ± standard deviation, and compared with the normal range published by Charles River Laboratories (n = 3). All the values that do not fall in normal range are marked with the symbol*. (A–D) Part of comprehensive lipid and metabolic panel tests. Note: Only tests that show values outside the normal range for at least one animal group are included in Table 2. The results of all other tests are included in the supplementary information Table S3. Abbreviation; ALP: Alkaline Phosphatase; ALT: Alanine amino transferase; TRIG: Triglyceride.
