# Supplementary material for: Towards An Advanced Graphene-Based Magnetic Resonance Imaging Contrast Agent: Sub-acute Toxicity and Efficacy Studies in Small Animals
Source: Sci Rep. 2015 Dec 2;5:17182. doi: 10.1038/srep17182 (PMC4667281; doi:10.1038/srep17182)
Supplement: Supplementary Information [file srep17182-s1.pdf]

Supplementary Information for

Towards An Advanced Graphene-Based Magnetic Resonance Imaging Contrast Agent: Sub-acute Toxicity and Efficacy Studies in Small Animals

Shruti Kanakia[1], Jimmy Toussaint[1], Dung Minh Hoang [2], Sayan Mullick Chowdhury[1], Stephen Lee[1] BS, Kenneth R. Shroyer[3], William Moore[4], Youssef Z. Wadghiri[2*], Balaji Sitharaman[1*]

[1]Department of Biomedical Engineering, [2]Department of Radiology, Bernard & Irene Schwartz Center for Biomedical Imaging, NYU School of Medicine, New York, NY, [3] Department of Pathology, [4]Department of Radiology, Stony Brook University, Stony Brook, NY, USA

*Address Correspondence to:

Balaji Sitharaman, Ph.D., Department of Biomedical Engineering, Bioengineering Building, Rm #115, Stony Brook University, Stony Brook, NY 11794-5281, USA; Ph: 631-632-1810, Email: balaji.sitharaman@stonybrook.edu

and

Youssef Z. Wadghiri, Ph.D., Department of Radiology, Bernard & Irene Schwartz Center for Biomedical Imaging, NYU School of Medicine, 660 First Avenue 4th floor, Room 444 New York, NY 10016, USA; Ph: 212 263 3336, Email: Wadghiri@med.nyu.edu

Tables

Table S1: Physicochemical characterization and *in vitro* study results of Mangradex formulation.

Abbreviations: TEM, transmission electron microscopy; AFM, atomic force microscopy; EELS, electron energy loss spectroscopy; RS, Raman spectroscopy; ICP-MS, inductively coupled plasma mass spectrometry; EPR, electron paramagnetic resonance; SEM, scanning electron microscopy; TGA, thermal gravimetric analysis.

Study/ Reference	Condition	Duration	Evaluation Method	Noteworthy Findings
Particle size	Mangradex		TEM, AFM	Size of graphene Nanoplatelets ~20-40 nm, Thickness 3-4 nm
Size distribution	Mangradex		TEM	100±20 nm
Dispersibility	Mangradex	0.4, 10, 2, 50, 100, 200 mg/mL	Visual assessment	Stable colloidal dispersion up to 100 mg/ml.
Structure/shape,	Mangradex		TEM, AFM	Disc shaped particles
Chemical composition	Mangradex		EELS, RS, ICP-MS,EPR	
Topology	Mangradex		TEM,SEM, AFM	Dextran coils around the graphene nanoparticles
Quantitative composition	Mangradex		ICP-MS<br>TGA	GNP-60% by weight.<br>Dextran-40% by weight.<br>Manganese – 0.064 % by weight.
Surface coating and composition	Mangradex		TGA	Dextran 10K; 40% by weight
Stability - chemical-thermal stability	Mangradex 20, 50, and 100 mg/mL	3 and 24 hours, 30 days @ 25°C and 37°C	$NaBiO_3$ test- UV-Vis spectrophotometer	No color change observed in sodium bismuthate ($NaBiO_3$) test. Negligible < Limit Of Detection (0.01µM) of UV-Vis

				spectrophotometer.
Dispersion stability	Mangradex 0.4, 10, 20, 50, and 100 mg/mL in Distilled Deionized water + mannitol (55 mg/ml)	0, 1, 2, 4, 24 hours after formulation preparation	Visual assessment and digital photograph of the solution	No apparent precipitation through 4 hours - all concentrations. Apparent precipitation at 100 mg/kg at 24 hour, but easily redispersed.
Osmolality	0.4-100 mg/mL in DDI water	*In vitro*	Osmometer	Hypo-osmolar in DDI water (2-186 mOsm).<br>With addition of Mannitol in Mangradex solution roughly iso-osmolar range to blood (190-320 mOsm/kg).
Viscosity	0.4-100 mg/mL	*In Vitro*	Viscometer	1.01-3.81 cP @ 37°C, 50 rpm and 0.86-2.09 cP @ 37°C, 100 rpm; values similar to blood viscosity (3-4 cp) and generally other approved MRI agents.
Hydrophilicity/partition co-efficient	20 mg/mL	*In Vitro*	Flask shaking method and analysis of nanoparticle concentration by spectro-photometry	Very hydrophilic with low partition coefficient; log K_{ow} = -0.18.

Table S2: *In vitro* and *in vivo* studies of Mangradex formulation.

Abbreviations: ELISA,enzyme-linked immunosorbent assay; OPT, o-phthalaldehyde**;** Rxn, reaction; PEI, Polyethylene imine; RBC, red blood cells; ACh, acetylcholine; ADO, adenosine; PE, phenylephrine; PF4, platelet factor 4; Sc5b9, Soluble terminal Complement Complex; Bb, factor B.

**Study/ Reference**	**Species/ Test System**	**Condition**	**Duration**	**Route**	**Evaluation**	**Noteworthy Findings**
Protein Binding	Human albumin	0.1,1 and 10 mg/mL	37°C for 24 hours	*In vitro*	Equilibrium dialysis and UV-Vis spectrophotometer	Mangradex did not show significant binding to protein.
Histamine Release	Whole blood, human	0.1, 1, and 10 mg/mL – Mangradex	60 min at 37°C	*In vitro*	Histamine levels (test kit) – ELISA	Negligible ↑ in level of histamine release up to 0.1 mg/mL when compared with controls.
	Mast cells, rat	0, 1, 7, and 10 mg/mL Mangradex	5 min pre-treatment		Histamine-OPT Rxn – Fluorescence intensity	Negligible ↑ in level of histamine release up to 10 mg/mL when compared with controls.
Platelet Activation	Whole blood, human – 2 donors	0, 1, 7, and 10 mg/mL Mangradex	45 min pre-treatment	*In vitro*	PF4 release - ELISA	Negligible ↑ in level of PF4 up to 10 mg/mL when compared with controls.

Study/ Reference	Species/ Test System	Condition	Duration	Route	Evaluation	Noteworthy Findings
Complement Activation	Whole blood, human- 2 donors	0, 1, 7, and 10 mg/mL Mangradex	45 min pre-treatment	*In vitro*	Sc5b9 or Bb levels (test kit) – ELISA	No significant ↑ in Sc5b9 at 1 and 7 mg/mL Mangradex compared with controls.<br><br>Significant ↑ (≈20% compared with controls) at 10 mg/mL.<br><br>Similar ↑ (≈18%) in Bb at 4 mg/mL dextran but not at 0.4 or 2.8 mg/mL when compared with controls.<br><br>↑ in complement activation at high concentration likely a function of dextran.
RBC Aggregation	Whole blood, human- 1 donor	0, 1, 7, and 10 mg/mL Mangradex<br><br>Positive control - PEI	45 min pre-treatment	*In vitro*	Visualization (630X) of blood smears	No aggregation or morphological change in RBCs up to 10 mg/mL.<br><br>PEI caused aggregation.

Study/ Reference	Species/ Test System	Condition	Duration	Route	Evaluation	Noteworthy Findings
Vasoactivity	Hamster ; n = 8M/Mangradex ; 3M/dextran)	Vasoactivity<br>Mangradex – 0, 0.1, 0.5, 2.6, 10, and 50 mg/mL<br>Dextran – 3.5, 35 mg/mL		IV	Cheek pouch model – vasoactivity; ACh, ADO, PE response modification<br>- arcade and terminal arterioles junction - Δ in vessel diameter in response to vasoactive compounds	a Concentration dependent dilation with Mangradex; significant ↑ ≥ 2.4 mg/mL; EC50 = 2.4-2.6 mg/mL<br>with maximum dilation ranging from 60% (arcade arterioles) to 76% (terminal

Study/ Reference	Species/ Test System	Condition	Duration	Route	Evaluation	Noteworthy Findings
		Vasoresponse change – <br>Mangradex - 50 mg/mL; pre and 15-min post vasoactive agent exposure <br>4-10M ACh, ADO, PE				rterioles); no effect with dextran only. <br>No apparent effect on vasoresponse to ACh, ADO, PE at doses of 50 mg/mL. <br>Conclusion: No endothelial dysfunction associated with exposure to Mangradex based on an absence of alteration in response to vasoactive agents.

Table S3: Blood chemistry results for rats injected with Mangradex, dextran, or mannitol. Also included are sham controls. The data are shown as mean values ± standard deviation, and compared with the normal range published by Charles River Laboratories (n=3).

	Test	Sex	Normal	Sham	Mannitol	Dextran	1 mg/kg	50 mg/kg	100 mg/kg
A	WBC (K/µL)	M	1.9-11.1	5.5±0.2	6.6±2.5	6.9±1.8	4.9±0.8	7.1±1.9	9.7±1.7
		F	0.9-7.9	4.9±1.8	7.4±1.7	5.2±4.5	4.4±0.6	4.4±1.1	3.0±0.7
B	RBC (M/µL)	M	7.6-9.9	7.6±0.2	7.9±0.1	7.7±0.4	6.7±1.9	7.5±0.2	6.7±0.5
		F	7.2-9.2	7.6±0.2	7.7±0.2	7.7±0.5	7.6±0.6	6.8±0.2	6.1±0.5
C	Hemoglobin (g/dL)	M	13.6-17.4	15.5±0.6	14.9±0.1	14.7±0.6	14.8±1.3	14.5±0.3	13.3±0.5
		F	13.7-17.2	15.1±0.9	14.4±0.4	14.0±0.5	14.9±0.2	13.8±0.4	12.8±1.4
D	Hematocrit (%)	M	38.5-52.0	46.1±0.6	45.9±0.1	44.1±1.1	42.8±6.4	43.2±0.6	40.5±1.4
		F	38.5-49.2	44.5±2.4	43.4±0.1	42.2±1.5	44.6±0.4	40.2±0.8	38.2±2.7
E	Platelet Count (K/µL)	M	574-1253	579±60.8	540±181.1	776±181.8	898±113.0	1153.6±389	1194±303.1
		F	599-1144	422.6±127.7	612±87.1	674.7±51.8	930.5±53.0	972.3±197.8	894±213.7
F	MCV (FL)	M	46-56	60.4±0.8	58.5±0.9	57.4±1.1	58.8±2.7	57.7±0.6	60.6±4.1
		F	50-57	56.9±2.7	56.5±0.4	54.4±1.7	58.6±4.9	58.8±0.9	62.6±2.6
G	MCH (pg)	M	16.3-19.5	20.2±0.3	19.1±0.5	19.2±0.3	23.2±5.5	19.4±0.2	19.9±1.1
		F	17.6-20.3	19.1±0.9	19.2±0.6	18.1±0.6	19.6±1.7	19.9±0.2	20.8±0.8
H	MCHC (g/dL)	M	31.9-38.5	33.5±0.9	32.5±0.3	33.4±0.1	39.7±10.9	33.6±0.1	32.9±0.4
		F	33.2-37.8	33.6±0.3	34±0.8	33.2±0.1	33.4±0.3	33.9±0.3	33.3±0.3
I	RDW (%)	M	11.6-16.2	13.5±0.1	12.7±0.4	14.3±0.3	12.5±0.4	13.6±0.6	15.3±1.1
		F	10.6-14.6	12.7±1.3	11.9±0.2	9.2±6.9	12.2±1.5	12.8±0.3	15.2±0.9
J	MPV (FL)	M	6.1-9.5	5.8±0.1	6.1±0.1	5.9±0.2	6.0±0.3	6.3±0.1	6±0.1
		F	6.4-9.5	6.8±0.1	5.6±0.6	5.6±0.1	5.9±0.2	6.2±0.3	6±0.2
K	BUN (mg/dl)	M	10.7-20	22.7±1.5	22±1.4	19.7±1.2	19.5±2.4	22.3±2.8	19.7±2.6
		F	11.7-25	19.8±2.2	20.5±3.1	19±3.6	21±4.3	18.5±0.6	18±3
L	Creatinine (mg/dl)	M	0.3-0.5	0.2±0.05	0.2±0.01	0.2±0.03	0.2±0.05	0.2±0.1	0.2±0.1
		F	0.3-0.6	0.3±0.03	0.2±0.1	0.3±0.03	0.25±0.02	0.3±0.1	0.3±0.1
M	AST (U/L)	M	63-175	130.3±33.5	83±16.9	104.3±21.7	103.3±45.7	136.5±95.0	80.7±5.0
		F	64-222	142±54.9	117.3±35.9	121±23.1	96±5.7	85±18.9	122.3±21.4
N	Albumin (g/dL)	M	3.6-4.7	4.5±0.1	4.5±0.1	4±0.2	4.3±0.2	4.2±0.3	3.9±0.4
		F	3.7-5.8	4.8±0.3	4.5±0.2	4.9±0.1	4.6±0.1	4.9±0.2	4.6±0.3

O	CHOL (mg/dl)	**M**	37-95	89±11.5	82.5±7.8	74.7±10.7	82.8±8.1	97.5±21.3	93.5±14.4
		**F**	23-97	78.3±11.5	81±11.6	79.3±19.4	78±1.4	98±9.9	81.7±5.0
P	TP (g/dL)	**M**	5.6-7.6	6.8±0.29	6.7±1.2	6.3±0.4	6.5±0.3	6.7±0.2	6.6±0.4
		**F**	5.7-8.3	6.9±0.4	6.9±0.4	7.0±0.1	6.8±0.0	7.1±0.6	6.7±0.1
Q	Na (mmol/L)	**M**	137-147	146.3±2.1	145±1.4	139.7±1.2	144±4.6	146±0.8	148.3±4.9
		**F**	135-146	145.8±1.5	150.5±6.1	143.7±1.5	149.5±2.1	146.5±3.4	146.3±1.5
R	K (mmol/L)	**M**	3.9-6.1	6.6±1.5	6.4±0.9	5.5±0.5	6.3±1.3	5.5±1.9	7.6±2.1
		**F**	3.4-5.1	6.5±0.8	6.8±1.9	5±0.9	5.1±0.2	5.1±0.7	5.9±0.2
S	Cl (mmol/L)	**M**	98-116	97±0.0	96.5±0.7	93.7±0.6	97.3±2.2	98.0±0.6	99.5±.1.9
		**F**	97-106	99.5±2.9	99.5±4.2	97.7±2.3	103.5±3.5	99.5±1.3	100±1.0
T	CO_2 (mmol/L)	**M**	-	34.7±8.4	39±9.9	34.3±1.5	31.5±3.1	30.5±5.5	24.3±9.9
		**F**	-	29.5±4.8	30.5±6.81	37.7±1.53	24±14.14	32.8±3.77	33.7±4.9
U	Ca (g/dl)	**M**	9.1-11.9	13.5±0.5	13.4±1.9	12.1±0.2	12.9±0.8	13.8±0.9	12.4±0.7
		**F**	9.5-12.1	13.2±0.9	12.3±0.8	11.8±0.5	12.1±0.4	12.6±0.6	12.7±0.4

(A-J) Part of complete blood count tests, and (K-U) part of comprehensive lipid and metabolic panel tests. Abbreviation - WBC: White blood cell, RBC: Red blood cell, MCV: Mean corpuscular volume; MCH: Mean corpuscular hemoglobin, MCHC: Mean corpuscular hemoglobin concentration, RDW: Red cell distribution width, MPV: Mean platelet volume, BUN: Blood urea nitrogen AST: Aspartate amino transferase, TP: Total protein, CHOL: Cholesterol

Figure Legends

Figure S1. A) Representative transmission electron microscope image of Mangradex. **B)** Digital image of Mangradex formulation at 20, 10 and 0.4 mg/ml used for sub-acute toxicity study at 100, 50 and 1 mg/kg dose respectively.

Figure S2: Body weights of the rats were measured 3 times/ week before injection during the sub-acute toxicity study. **A)** Body weights of the female rats **B)** Body weights of the male rats. Values are mean ± S.D. One-way Anova shows no statistically significant differences ($p < 0.05$) among the groups.

Figure S3: Blood pressures of the rats were measured once at the beginning of the week during the sub-acute toxicity study. **A)** blood pressure of the female rats **B)** blood pressure of the male rats. Values are mean ± S.D. One-way Anova shows no statistically significant differences ($p < 0.05$) among the groups.

Figure S4: Heart rates of the rats were measured once at the beginning of the week during the sub-acute toxicity study. **A)** heart rate of the female rats **B)** heart rate of the male rats. Values are mean ± S.D. One-way Anova shows no statistically significant differences ($p < 0.05$) among the groups.

Figure S1

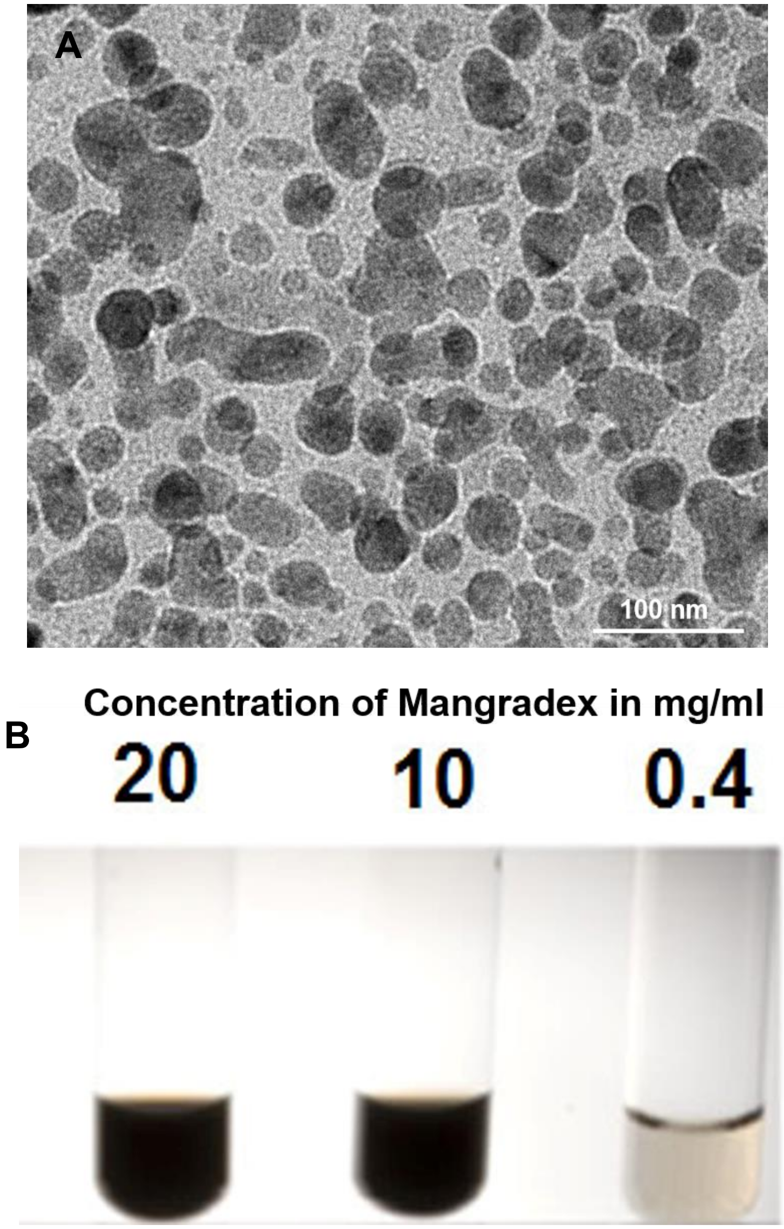

Figure S2 A

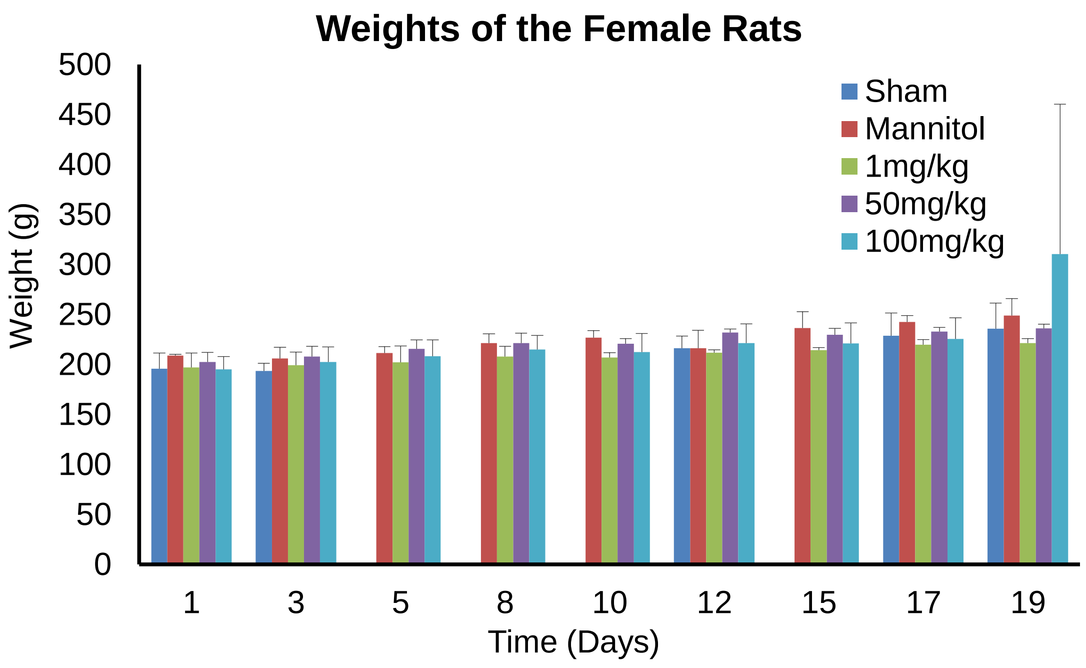

Weights of the Female Rats
500
450
400
350
300
250
200
150
100
50
0
Weight (g)
Sham
Mannitol
1mg/kg
50mg/kg
100mg/kg
1
3
5
8
10
12
15
17
19
Time (Days)

Figure S2 B

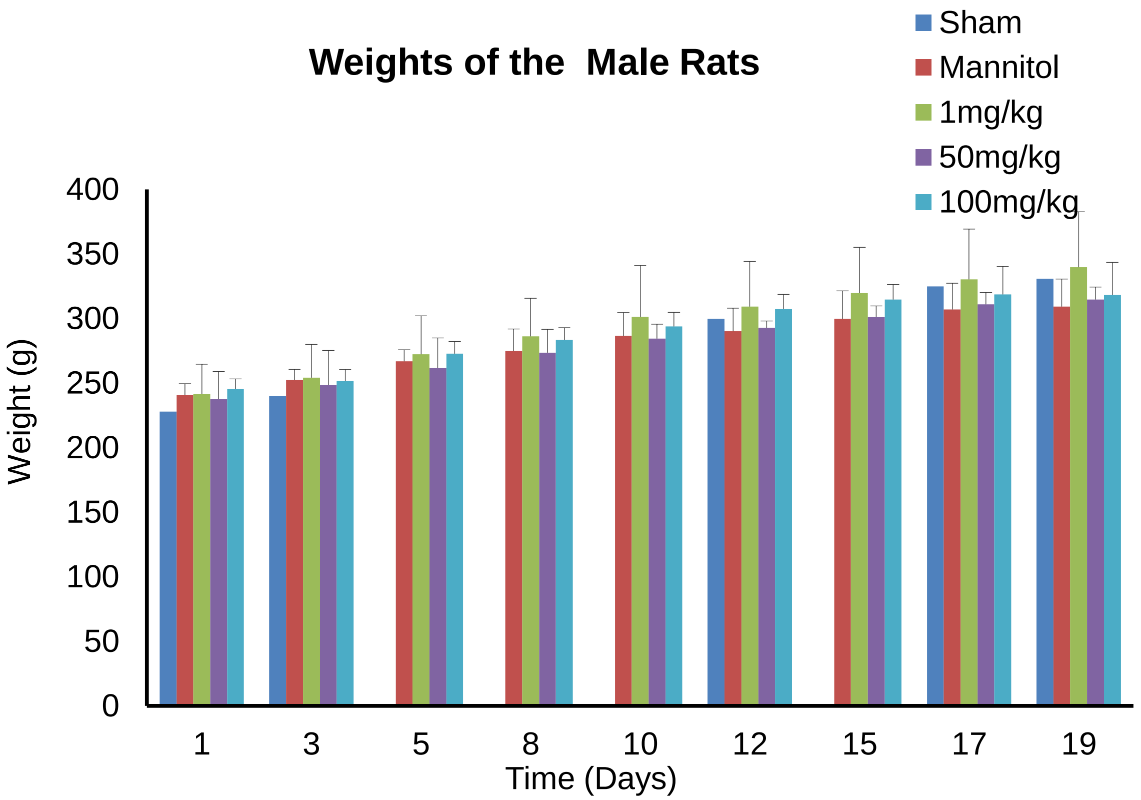
Weights of the Male Rats
Sham
Mannitol
1mg/kg
50mg/kg
100mg/kg
Weight (g)
0
50
100
150
200
250
300
350
400
1
3
5
8
10
12
15
17
19
Time (Days)

Figure S3 A

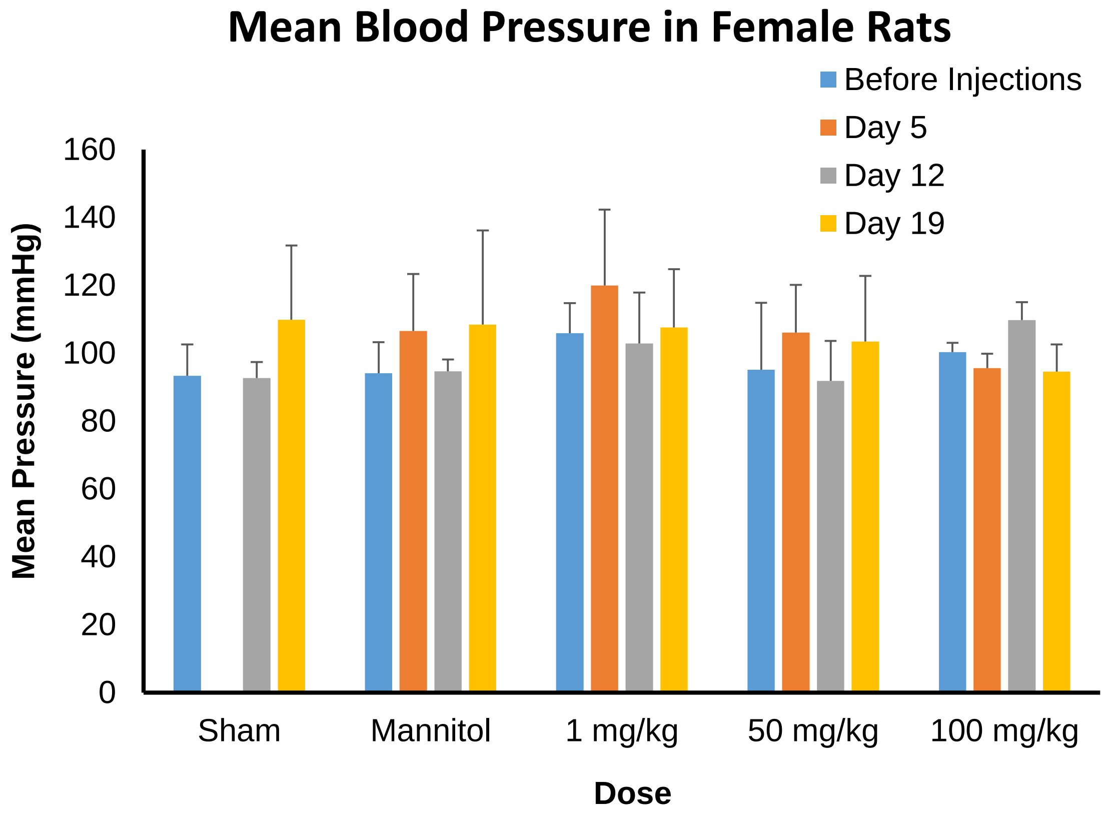
Mean Blood Pressure in Female Rats
Before Injections
Day 5
Day 12
Day 19
Mean Pressure (mmHg)
0
20
40
60
80
100
120
140
160
Sham
Mannitol
1 mg/kg
50 mg/kg
100 mg/kg
Dose

Figure S3 B

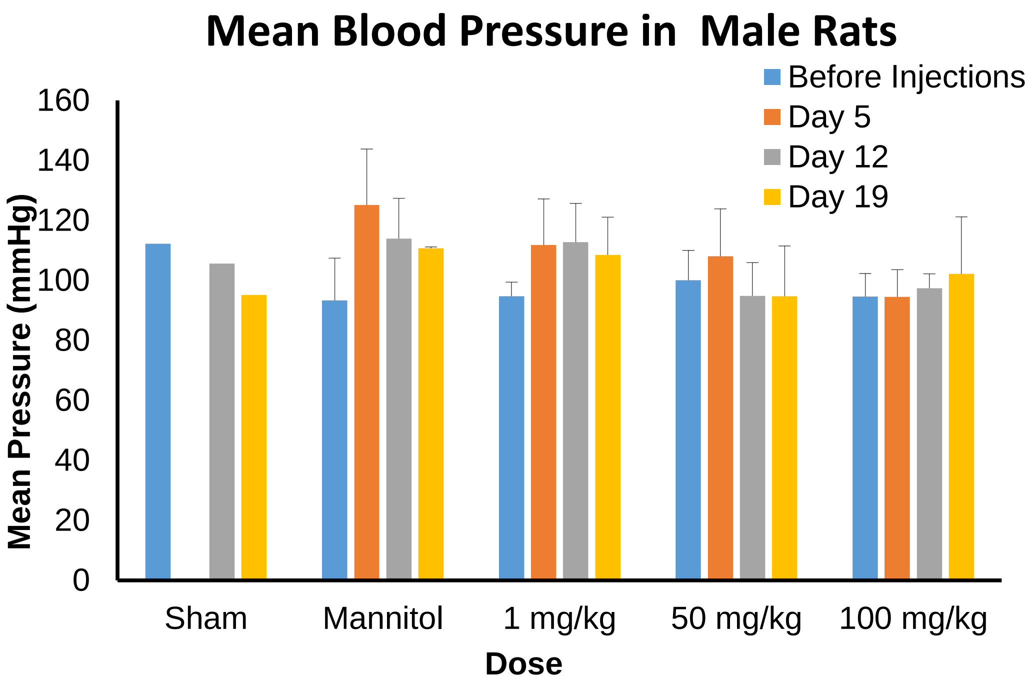
Mean Blood Pressure in Male Rats
Before Injections
Day 5
Day 12
Day 19
Mean Pressure (mmHg)
160
140
120
100
80
60
40
20
0
Sham
Mannitol
1 mg/kg
50 mg/kg
100 mg/kg
Dose

Figure S4 A

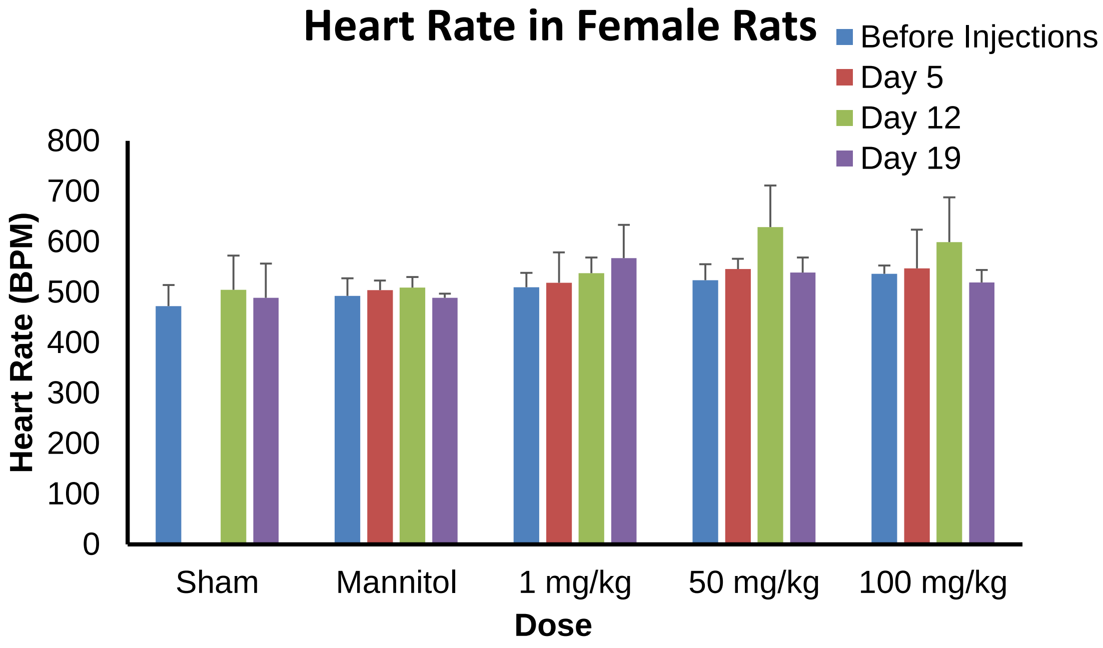

Heart Rate in Female Rats
Before Injections
Day 5
Day 12
Day 19
Heart Rate (BPM)
800
700
600
500
400
300
200
100
0
Sham
Mannitol
1 mg/kg
50 mg/kg
100 mg/kg
Dose

Figure S4 B

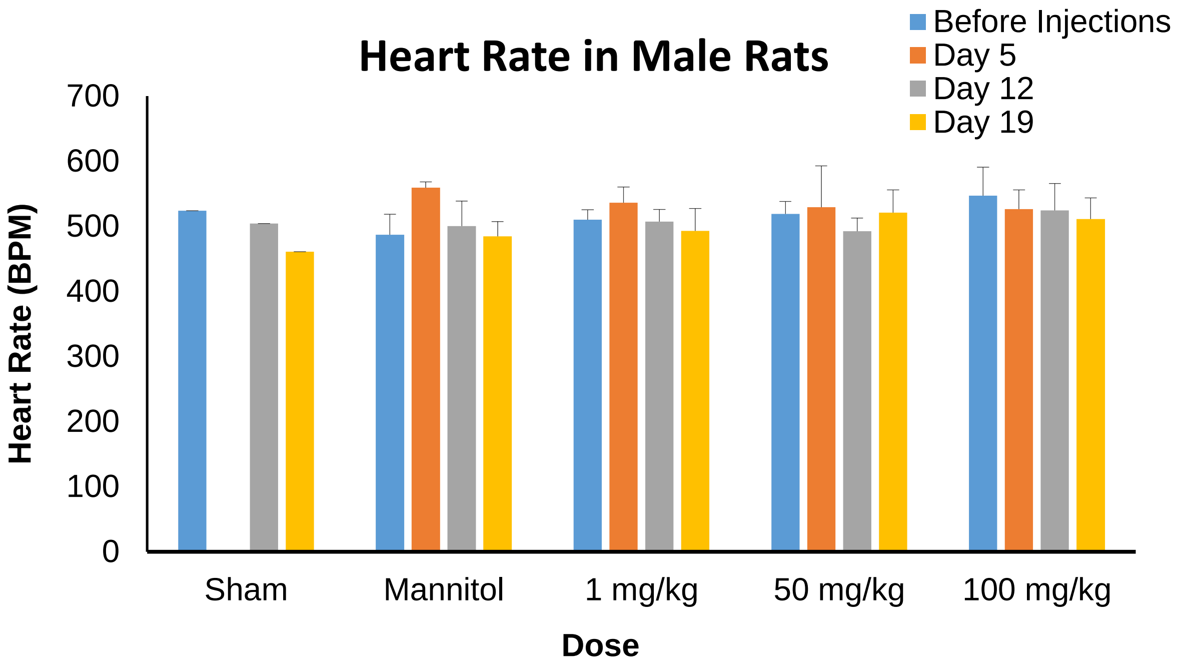
Heart Rate in Male Rats
Before Injections
Day 5
Day 12
Day 19
Heart Rate (BPM)
700
600
500
400
300
200
100
0
Sham
Mannitol
1 mg/kg
50 mg/kg
100 mg/kg
Dose
